# Case of Acute Renal Dropsy

**Published:** 1871-05

**Authors:** 


*Case of Acute Renal Dropsy.* Under the care of DR. HENRY THOMPSON.

(British Medical Journal, August 6.)

At a meeting of the Pathological Society of London, held May 17th, Dr. Cayley brought forward a case of acute renal dropsy without albuminuria, with interstitial nephritis. The patient, a boy aged nine, was a patient in the Middlesex Hospital, under the care of Dr. Henry Thompson. His previous health had been good, and he had never had scarlatina. About fourteen days before his admission he caught cold, and seemed generally unwell. A few days afterwards his throat became sore, and this was shortly after followed by a purulent discharge from both ears. No rash was observed; and, so far as could be ascertained, he had not been exposed to scarlatinal infection. His brothers and sisters also remained well. About a week after the accession of his symptoms, general dropsy supervened, beginning in the eyelids and face; this continued to increase, and during the two nights preceding his admission he was delirious. The condition of his urine was not noticed. He does not appear to have suffered from pain in the loins. On admission he was well nourished, but had a pasty anæmic complexion. There was considerable general anasarca, and some degree of ascites. Pulse 120; respirations 24. The thoracic percussion and respiratory sounds were normal. A soft double murmur was audible over the præcordia, most distinct at the base of the heart. There was a purulent discharge from the ears; but the sore throat had almost subsided. No desquamation was present. The urine was not high-colored, of specific gravity 1018; it contained a deposit of lithates, dissolved by heat. On continuing to apply heat, a white cloud formed, redissolved by nitric acid. On adding nitric acid to the cold urine, a deposit was thrown down, redissolved by heat. On microscopical examination, lithates and numerous large crystals of lithic acid were found, but no blood or casts. The urine was passed in rather large quantities, the specific gravity varying from 1012 to 1018. It was never found to contain either albumen, casts, or blood, but continued to show a considerable deposit of lithic acid. The dropsy much diminished, without completely disappearing. The patient was delirious at night. The heart's action became very irregular and tumultuous; and the double basis bellows-murmur, though altering much in character, was persistent. Ultimately, lobular pneumonia and œdema of the lungs supervened; and he died, after having been in the hospital eleven days, between three and four weeks from the commencement of his illness. He did not become comatose, and had no convulsions. On post-mortem examination, the lungs were found highly œdematous, with patches of lobular pneumonia dispersed through them. The heart showed recent myo and endo-carditis; the muscular walls of the left ventricle being studded with buff-colored patches, intermixed with points of extravasation. On microscopical examination of these patches, the muscular fibres were found thickly studded with oil-globules, and in some places appeared reduced to an oily and granular debris. The aortic valves were much swollen, softened, and studded with recent vegetations. The kidneys, especially the left one, were enlarged. The right one $4\frac{1}{2}$ oz.; the left $5\frac{1}{4}$ oz. Their capsules stripped off with abnormal facility; their surfaces were smooth, pale, but somewhat mottled. On section, the cortical parts were found increased in thickness; they were pale and somewhat opaque; the pyramids were congested. The kidneys, therefore, to the naked eye, presented much the characters of the large white kidney of the second stage of acute tubal nephritis. On microscopical examination, the condition was found to be very different. The morbid change consisted in the deposition of masses of nuclei, having the characters of lymph-corpuscles, between the uriniferous tubules and round the Malpighian capsules; they were especially abundant in the latter situation. The epithelium of the convoluted tubes appeared normal, or at the most was very slightly swollen and granular. The morbid condition was, therefore, essentially one of acute interstitial nephritis, the inflammatory exudation being in every respect identical with that met with in other interstitial inflammations—as, for example, cirrhosis of the liver. Cases of acute renal dropsy without albuminuria, both as the result of scarlatina and from exposure to cold, have been frequently recorded; but no very satisfactory explanation of their pathology appears to have been given. This case would tend to show that some of these perplexing cases, even when the result of scarlatina, as in all probability the present one was, may be due to interstitial nephritis. It is interesting also as an example of the acute stage of the contracted granular kidney, which is probably of rare occurrence. Certainly, in the great majority of cases of this form of Bright's disease, the nuclear deposit and its conversion into fibrous tissue go on with equal steps. The non-occurrence of albuminuria in the acute form and its late occurrence in the chronic form of the disease admit of a satisfactory explanation, on the supposition that the fluid which transudes from the Malpighian tufs, as they are not covered by any *secreting* cells, consists essentially of serum, and is therefore albuminous. In the healthy kidney the albumen and non-urinary constituents are reabsorbed during the passage of the fluid down the convoluted tubes. When the tubal epithelium is diseased, this reabsorption is interfered with, and albumen consequently appears in the urine. But in interstitial nephritis, which results in the contracted granular kidney, the renal epithelium does not become affected till the interstitial deposit, by its contraction, has begun to press upon it and constrict the convoluted tubes; and so albuminuria does not show itself till the disease has made considerable progress. The deposit also leads to thickening of the small arteries and capillaries; and this, while not interfering with the free transudation of water, tends to check the passage of the dissolved solids; and hence the urine in these cases is usually abundant and of low specific gravity. A similar condition of urine is found in cases where the vessels are thickened from other causes, as amyloid infiltration. The discovery of the termination of the convoluted tubes in Henle's loops speaks strongly in favor of this view, as this arrangement must necessarily greatly retard the flow of fluid down the convoluted tubes, and so favor reabsorption, while it would be unfavorable to secretion.

